# Indian major basmati paddy seed varieties images dataset

**DOI:** 10.1016/j.dib.2020.106460

**Published:** 2020-10-28

**Authors:** Arun Sharma, Deepshikha Satish, Sushmita Sharma, Dinesh Gupta

**Affiliations:** International Centre for Genetic Engineering and Biotechnology, Aruna Asaf Ali Marg, New Delhi 110067, India

**Keywords:** Images dataset, Basmati seeds, Deep learning, Classification, Indian basmati paddy varieties

## Abstract

The dataset contains images of 10 out of 32 notified Indian basmati seeds varieties (by the Government of India). Indian basmati paddy varieties included in the dataset are 1121, 1509, 1637, 1718, 1728, BAS-370, CSR 30, Type-3/Dehraduni Basmati, PB-1 and PB-6. Moreover, several images of other seeds and related entities available in the household have also been included in the dataset. Thus, the dataset contains 11 classes such that ten classes contain images from ten different basmati paddy varieties. In contrast, the 11th class- named “Unknown” contains images from a mixture of two morphologically similar paddy varieties (1121 and 1509), different pulses, other grains and related food entities. The Unknown class is useful in discriminating the paddy seeds from other types of seeds and related food entities. All the images were captured (in standard conditions) manually using an apparatus developed *in-house* and a tablet with a five-megapixel camera (5MP). The camera was used to capture 3210 RGB coloured images in JPG format. The data pre-processing was performed to generate the ready-to-use images for training and testing machine learning-based models. AI-based paddy seed variety classification models have been developed using the dataset. The dataset can be used to generate different types of AI-based models for adulteration detection, automated classification models (along with independent devices) at the time of rice threshing, and to increase the classification potential (Supplementing images representing additional basmati varieties).

## Specifications Table

**Table:** 

Subject	Artificial Intelligence
Specific subject area	Development of Artificial Intelligence-based major basmati paddy-seed variety classification models.
Type of data	Image
How data were acquired	A customized apparatus was designed and used to place paddy seed grains. After that, a tablet with a 5 megapixel (MP) camera was used to capture the images (in standard conditions) of these paddy seed grains. Further, data pre-processing was done to retrieve the images required as input for the training and testing of machine learning models.
Data format	Filtered (.jpg)
Parameters for data collection	Apparatus dimensions: 33.5 cm * 20 cm * 11 cm (Length* Breadth * Height *) Background: White Area of well: 8.5 cm * 8.5 cm * 1 cm Light Source: Two LED bulbs (4 watts each), diagonally placed, crystal white, luminous flux 370 lumens. Camera Specification: Micromax Canvas TAB P802 (5MP). Aperture length: f/2.8 Focal length: 3.5 Metering mode: Centre-weighted average. Type of Images: RGB coloured (2560 × 1920 pixels; JPG format).
Description of data collection	Ten different samples of paddy seeds of different varieties were collected from the Indian Agricultural Research Institute (IARI), New Delhi, India. Of these ten varieties, five varieties seeds, i.e., 1121, 1509, 1637, 1718 and 1728, were collected from the Seeds Production Unit, while the remaining five varieties seeds, i.e., BAS-370, CSR 30, Type-3/Dehraduni Basmati, PB-1 and PB-6) were provided by Genetics Department, IARI, New Delhi, India. After that, a customized apparatus was designed and loaded with sample seeds. A Micromax Canvas TAB P802 tablet with a 5MP camera was used to capture the images.
Data source location	Institution: International Centre for Genetic Engineering and Biotechnology (ICGEB) City: New Delhi Country: India
Data accessibility	All the images have been uploaded on an open, free-to-use research data repository named “Mendeley Data.” The specific details to access the images data are: Sharma, Arun; Satish, Deepshikha; Sharma, Sushmita; Gupta, Dinesh (2020), “Indian Major Basmati Paddy Seed Varieties Images Dataset,” Mendeley Data, V2 http://dx.doi.org/10.17632/byms2fjzpw.2 Repository name: Mendeley Data Data identification number: 10.17632/byms2fjzpw.2 Direct URL to data: http://dx.doi.org/10.17632/byms2fjzpw.2 Instructions for accessing these data: The dataset is publicly accessible from the above mentioned URL.
Related research article	A. Sharma, D. Satish, S. Sharma, D. Gupta, iRSVPred: A Web Server for Artificial Intelligence Based Prediction of Major Basmati Paddy Seed Varieties. Front Plant Sci. (2020)1791. https://doi.org/10.3389/fpls.2019.01791

## Value of the Data

• The images dataset can be instrumental in the automatic or AI-assisted classification of paddy varieties of global economic importance.
• Indian Basmati rice is of high commercial value. In the financial year 2018–19, Indian basmati accounted for over 90% of the overseas basmati rice market [1]. The data will be helpful to basmati paddy growers, basmati paddy-seeds importers and exporters, government, rice mills, etc.
• The images dataset can be used to develop more accurate and other types of AI-based classification models. For example, basmati paddy adulteration detection models and development of independent devices for automatic quality check of basmati paddy grains (at a larger scale) at rice threshing mills.
• At present, images have been generated for only 10 basmati paddy varieties while total 32 notified varieties of basmati are reported in literature [2]. Thus, new images can be generated for the remaining varieties and existing images can be used for new AI-based models training and validation (to classify other basmati paddy varieties).

## 1 Data Description

Seeds from ten major Indian basmati paddy varieties were collected from the Indian Agricultural Research Institute (IARI), New Delhi, India [2]. A total of 46 different types of pulses, grains and other food entities were also collected *in-house* to capture images other than paddy seeds. Moreover, a mixture of two morphologically similar paddy varieties (1121 and 1509) was also prepared in a separate vessel and its images constituted a new class (including pulses, grains and other food entities). Thus, the dataset (accessible on Mendeley) comprises 11 classes (10 basmati paddy seeds varieties and other grains and related food entities). An apparatus was designed to capture the images in standard conditions. A Micromax Canvas TAB P802 tablet, attached to the apparatus, was used to capture 3210 images (Table 1).

**Table 1:** The variety-wise distribution of images in the dataset.

S. No.	Variety	Number of Images taken
1	Basmati 370	250
2	Type 3 (Dehraduni Basmati)	250
3	Pusa Basmati 1	250
4	Pusa Basmati 1121	250
5	Pusa Basmati 6 (Pusa 1401)	250
6	Basmati CSR 30	250
7	Pusa Basmati 1509 (IET 21960)	250
8	Pusa Basmati 1637	250
9	Pusa Basmati 1728	250
10	Pusa Basmati 1718	250
11	Mix varieties (1121 plus 1509)	250
12	Other seeds and related entities	460
	Total Images	3210

## 2 Experimental Design, Materials and Methods

*Collection of Seeds:* Ten Indian basmati paddy varieties seeds were collected from the Indian Agricultural Research Institute (IARI), New Delhi. Of these, five seed varieties- 1121, 1509, 1637, 1718 and 1728 were collected from the Seeds Production Unit, while the remaining five varieties seeds- BAS-370, CSR 30, Type-3/Dehraduni Basmati, PB-1 and PB-6) were obtained from the Genetics Department, IARI, New Delhi, India.

**Fig. 1:**
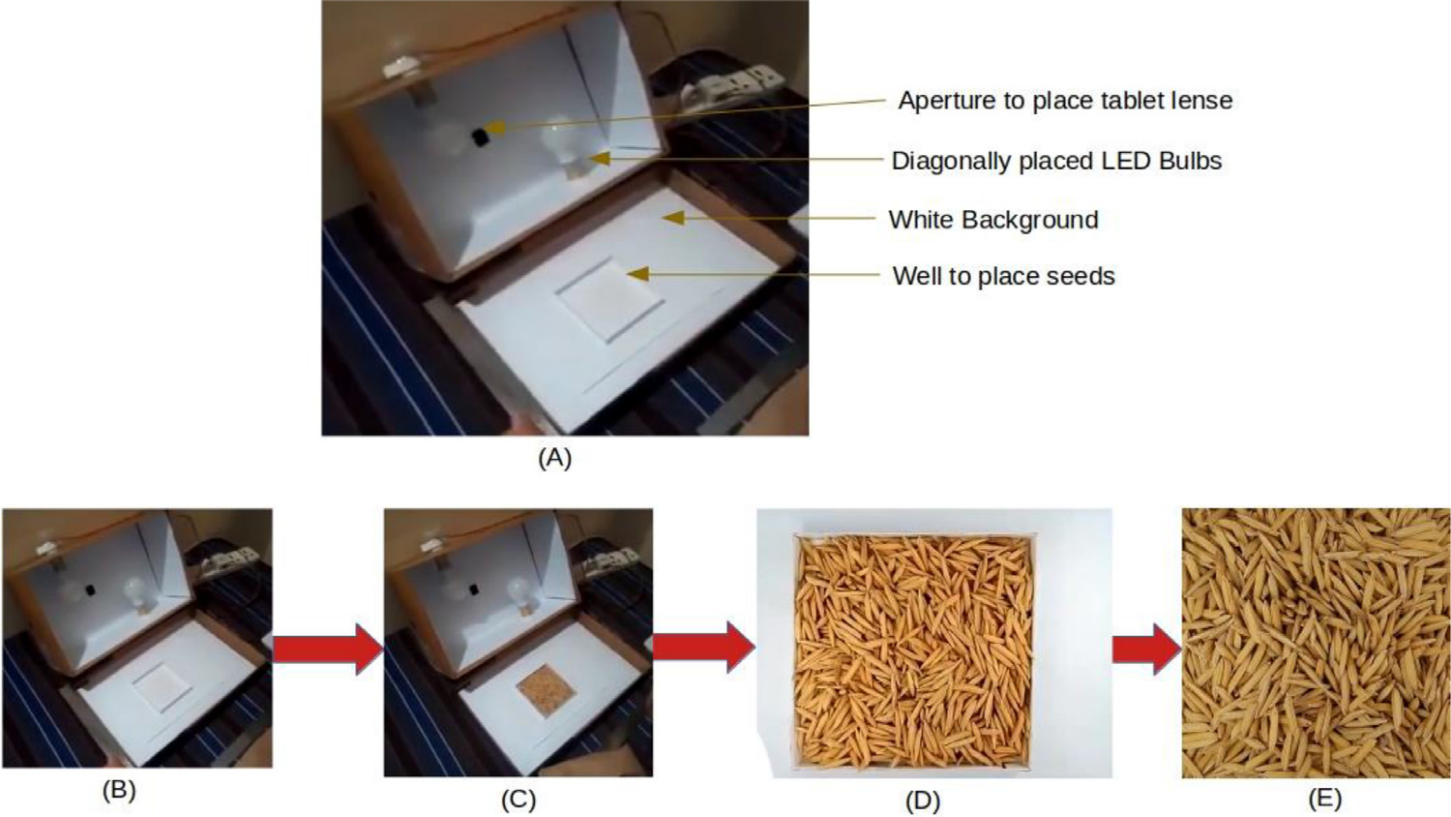
Apparatus designed and used to capture images of major Indian basmati paddy seeds varieties.

*Apparatus Designed to Capture Images:* An apparatus with dimensions of 33.5 cm * 20 cm * 11 cm (Length* Breadth * Height) was designed to capture the images in a controlled condition (Fig. 1). As shown in Fig. 1, a cardboard material was used to build the image capturing apparatus. Plain white paper was used to make the background inside colour white, and a small aperture was created to place a tablet lens over it while capturing the images. Two LED bulbs (4 Watts each, crystal white, luminous flux 370 lumens) were fitted diagonally to provide white light and cancel any shadow effect.

The bulbs rendered artificial lighting conditions that helped in capturing good quality images (for each image). A small well type structure of dimensions 8.5 cm * 8.5 cm * 1 cm (length*breadth*depth), was created to place samples of the seeds in a fixed area (ensuring same dimensions for seeds of each variety type). Thus, the apparatus provided a standard conditions for capturing the paddy seeds images. Moreover, the apparatus can act as a general-purpose device or tool to capture the standard condition images that may be required for any other machine learning or AI-based study.

*Tablet and Lens Specifications:* A Micromax Canvas TAB P802 tablet with a five-megapixel camera (5MP) was used to capture the images. Initially, the camera captured RGB coloured images of size 2560 × 1920 pixels in the JPG format. For capturing the dataset images, an aperture of f/2.8, a focal length of 3.5 and centre-weighted average metering mode was used.

*Methodology Used to Capture the Images:* To capture the seed samples' images, several rounds of random sample selections were performed. For each round, a variety was selected and its sample placed inside the well, an image was captured (after focussing the camera on seeds) and images stored in the directories specific to each variety. Hence, multiple images of all the ten paddy varieties seeds were captured and stored in directories marked for each of the varieties. For each of the variety type, 250 images were captured. Thus, for all ten varieties, 2500 images have been captured (Table 1).

*Mix Varieties, Other Seeds and Related Entities Images:* In order to enable the AI-based models to identify and differentiate correct variety from the mixed varieties and other seeds or related entities, training with these types of datasets needs to be performed. Hence, to prepare such a dataset, two morphologically highly similar varieties (1121 and 1509) seeds were mixed in a separate vessel and 250 different images were captured. Moreover, a total of 46 different types of seeds and related entities including pulses, grains, etc., were also collected *in-house* and used to capture 460 images (10 images for each seed type and associated entities). Therefore, mixed varieties of images and other seeds and related entities collectively constituted a new class type with 710 images. Thus, the images dataset comprises of 3210 images (2500 images from 10 basmati paddy varieties and 710 images from mixed varieties, along with other seeds and related entities) captured using the customized apparatus (Table 1).

*Images pre-processing:* For any machine learning or AI-based studies, data pre-processing is an essential step. Therefore, in order to prepare an ideal dataset and focus on seed images only, the boundaries of well comprising blank space (in captured images) were cropped using PERL scripts and ImageMagick software (ImageMagick 6.7.8-9) [3]. The modified images of size (1450 × 1450) have been finally provided, which are ready for the training and validation of the AI-based prediction models.

*Division of Images Dataset for AI-based studies:* To facilitate the direct usage of images for machine learning or AI-based studies, the dataset has been divided into three parts viz. (1) train (80%) (2) test (10%) and (3) validate or test_val (10%). The number of images in train, test and test_val datasets is 2568, 321 and 321, respectively.

## Ethics Statement

Not applicable.

## Declaration of Competing Interest

The authors declare that the research was conducted in the absence of any commercial or financial relationships that could be construed as a potential conflict of interest.
